# Analysis of the efficacy of hyperbaric oxygen therapy for disorders of consciousness: A retrospective cohort study

**DOI:** 10.1002/brb3.3588

**Published:** 2024-06-30

**Authors:** Sha Li, Zhi‐Juan Di, Zi‐Bo Liu, Long Zhao, Man‐Yu Li, Hong‐Ling Li

**Affiliations:** ^1^ Department of Rehabilitation The Second Hospital of Hebei Medical University Shijiazhuang China; ^2^ Department of Endocrinology The Second Hospital of Hebei Medical University Shijiazhuang China

**Keywords:** disturbance of consciousness, hyperbaric oxygen, influencing factors

## Abstract

**Objective:**

To analyze the efficacy and associated factors affecting the prognosis in patients with disturbance of consciousness after hyperbaric oxygen (HBO) treatment.

**Methods:**

A retrospective study was carried out on patients with disorders of consciousness (DOC) receiving HBO treatment from January to January 2022 in the Second Department of Rehabilitation Medicine of the Second Hospital of Hebei Medical University, China.

**Results:**

HBO therapy improved the Glasgow Coma Scale (GCS) and Chinese Nanjing Persistent Vegetative State Scale (CNPVSS), as well as the clinical efficacy in patients with DOC. The comparison of GCS and CNPVSS scores in patients with DOC before and after HBO treatment was all statistically significant, with 325 patients (67.1%) showing effective results and 159 patients (32.9%) having unchanged outcomes. Univariate analysis indicated that there were statistically significant differences in age, HBO intervention time, HBO treatment times, pre‐treatment GCS score, and etiology and underlying diseases between the good and poor prognoses groups. Multivariate regression analysis showed that HBO intervention time ≤7 days, HBO treatment ＞ times, high GCS score before HBO treatment, and brain trauma were independent influencing factors in achieving a good prognosis for patients with DOC. Low pre‐treatment GCS scores were an independent risk factor for a poor prognosis in patients with brain trauma while being male, late HBO intervention time, fewer HBO treatment times, and low pre‐treatment GCS scores were independent risk factors for a poor prognosis in patients with DOC after a stroke. Being ≥50 years of age, late HBO intervention time, and low pre‐treatment GCS scores were independent risk factors for a poor prognosis in patients with DOC after hypoxic‐ischaemic encephalopathy.

**Conclusion:**

HBO therapy can improve the GCS, CNPVSS scores and clinical efficacy in patients with DOC, and the timing of HBO intervention ≤7 days, times of HBO treatment, high pre‐treatment GCS score, and brain trauma were the independent influencing factors of good prognosis in patients with DOC.

## Graphical abstract

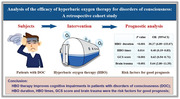

## 1 INTRODUCTION

Disorders of consciousness (DOC) are a group of conditions that impair the level of awareness and responsiveness of patients after brain injury. DOC can be caused by various etiologies, such as traumatic brain injury, stroke, and hypoxic‐ischaemic encephalopathy (HIE). Patients with DOC pose a huge economic burden and mental stress to their families and society (Eapen et al., 2017). According to foreign research findings, there are 100,000–300,000 patients with DOC in the United States, and the average prevalence of DOC is about 0.2–6.1 per 100,000 in Europe (Song et al., 2020). In China, due to the large population base and the impact of traditional moral concepts, few people give up treatment. There are about –100,000 new cases of DOC in China every year, and the total cost of treatment is about 30–50 billion RMB per year (Zhao, 2020). Therefore, finding effective treatments for DOC is an urgent and important issue.

Hyperbaric oxygen (HBO) therapy is a method of treating diseases by inhaling pure oxygen in an environment above atmospheric pressure. HBO can increase the oxygen supply to the brain, reduce intracranial pressure, relieve brain oedema, enhance anti‐inflammatory and anti‐apoptotic effects, and promote neurogenesis and angiogenesis (Y. Zhang et al., 2023). In recent years, HBO has been widely used in patients with DOC after brain trauma (Chen et al., 2022), stroke (Y. Wang et al., 2015), and HIE (Chen et al., 2021), but its efficacy has not been fully established, and there is no widely accepted set of guidelines for its use in these indications. These practices are generally considered “off‐label,” and most practitioners treat the limited existing data for HBO in these indications with caution. Moreover, the existing studies on HBO for DOC have some limitations, such as small sample sizes, the lack of a control group, short follow‐up periods, and inconsistent outcome measures (Hyldegaard & Hedetoft, 2020; Marcinkowska et al., 2022). Therefore, there is a need for more comprehensive and rigorous studies on the efficacy and safety of HBO for patients with DOC.

This study analyzed the efficacy and associated factors affecting the prognoses in patients with DOC after HBO treatment. To our knowledge, this is the largest and longest study on HBO for patients with DOC to date and provides valuable insights for clinical decision‐making and future research.

## 2 MATERIALS AND METHODS

### 2.1 Clinical data

#### 2.1.1 Research object

**FIGURE 1:**
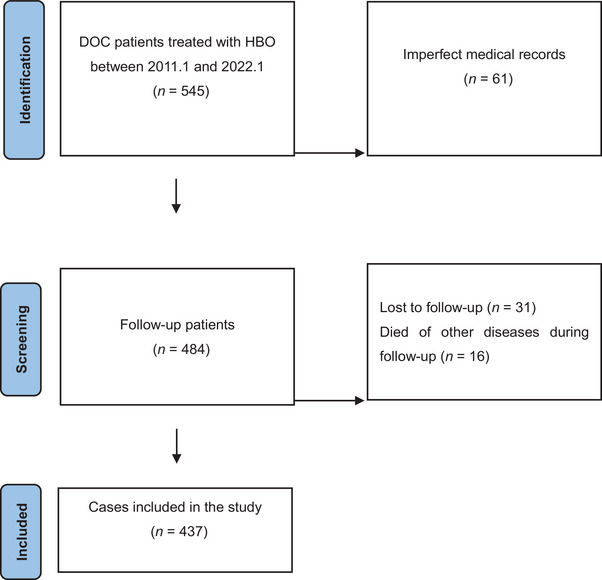
The flowchart of patient recruitment and dropout. DOC, disorders of consciousness; HBO, hyperbaric oxygen.

A total of 545 patients with DOC treated by HBO in the Second Department of Rehabilitation Medicine, the Second Hospital of Hebei Medical University from January 2011 to January 2022 were retrospectively analyzed. The flowchart of patient recruitment and dropout is shown in Figure 1, which was created using the PRISMA flow diagram generator. A total of 484 subjects were finally included for clinical characteristics and therapeutic effect analysis.

A total of 437 subjects were screened, and the influencing factors were analyzed. This study was approved by the ethics committee of the Second Hospital of Hebei Medical University and conducted in accordance with the Declaration of Helsinki. All patients or their legal guardians signed an informed consent form before participating in the study.

#### 2.1.2 Collecting data

Collecting basic information about all patients, gender, age, etiology, underlying disease, and patients’ Glasgow Coma Scale (GCS) and Chinese Nanjing Persistent Vegetative State Scale (CNPVSS) on admission and discharge, as well as clinical efficacy were evaluated. The basic information and prognoses of patients were followed up by telephone after discharge. Patients were divided into groups based on the Glasgow Outcome Scale (GOS), with those achieving 1–3 points representing the poor prognosis group and 4–5 points representing the good prognosis group.

#### 2.1.3 Inclusion criteria

The inclusion criteria for this study were as follows: (1) Patients with DOC with GCS scores below 8 points or conforming to a vegetative state (VS) and a minimally conscious state (MCS) diagnosis criteria (Kondziella et al., 2020); (2) patients with DOC arising from different causes, including brain trauma, stroke, and HIE; (3) the respiratory and circulatory system indexes and vital signs of the patient were stable; (4) course of disease was within 3 months; (5) all patients had first onset; (6) the total number of HBO treatments should not be fewer than 10.

#### 2.1.4 Exclusion criteria

The exclusion criteria for this study were as follows: (1) Patients with unstable vital signs; (2) patients with severe cardiac, liver, and renal dysfunction, hematological diseases and immune dysfunction; (3) patients with HBO contraindications, such as untreated pneumothorax, concurrent use of doxorubicin or disulfiram, chronic obstructive pulmonary disease or asthma with air‐trapping risk, recent ear surgery or injury, or claustrophobia; (4) patients who died during hospitalization due to other diseases; (5) patients with incomplete medical records.

#### 2.1.5 Instruments

The instruments used in this study were as follows:

1. Transcranial direct current stimulator: Model number: DK‐501; Manufacturing Company: Shijiazhuang Dukang Medical Device Co., Ltd.
2. Myoelectric biofeedback instrument: Model number: XCH‐B1; Manufacturing Company: Jiangxi Nuocheng Electric Appliance Co., Ltd.
3. EEG bionic electrical stimulator: Model: NK‐IA05; Manufacturing Company: Shijiazhuang Dukang Medical Device Co., Ltd.
4. Lower limb intelligent active and passive training system: Manufacturing Company: Beijing Baodahua Rehabilitation Equipment Technology Co., Ltd.
5. Vital Stim swallowing electric stimulator: Model number: 5900; Manufacturing Company: Beijing Chengkang Foundation Medical Equipment Co., Ltd.
6. Seven‐door air pressurized HBO chamber for medical No.3 cabin: Model: YC3200/0.3‐22VII; Manufacturing Company: Yantai Ice Ring Hyperbaric Oxygen Chamber Co., Ltd.

### 2.2 Methods

#### 2.2.1 Research grouping

Patients were divided into poor and good prognoses groups according to the GOS scores at follow‐up.

### 2.3 Treatment methods

All patients received routine monitoring of blood pressure, an electrocardiogram, and oxygen saturation, and they were given symptomatic treatment with drugs and underwent surgery according to their condition. Targeted rehabilitation treatment was given according to the specific dysfunction of the patients, including transcranial direct current stimulation, myoelectric biofeedback, neuroelectric biomimetic stimulation and the passive training of lower limb intelligence, and swallowing electrical stimulation. Based on these results, all of the patients received HBO treatment. The medical HBO three‐chamber seven‐door air‐pressurized chamber was used to deliver treatment at pressure ranges of 1.4–2.2 atmospheres absolute (ATA) (1.4–1.6 ATA for infants, 1.6–1.8 ATA for patients in a serious condition, the elderly, and the infirm, as well as for those with complications such as otorrhea and rhinorrhoea; 1.8–2.2 ATA was applied for stable patients).

The pressure was applied for 25 min until reaching the desired level. Then, pure oxygen was inhaled for 30 min, followed by a 10‐min air break and another 30‐min session of pure oxygen inhalation. Thereafter, the pressure was reduced over 25 min until returning to normal, once a day, 7 days per week, 30 times for each course of treatment, with a resting period of 5–7 days after each course of treatment.

### 2.4 Evaluation methods

We used the following methods to evaluate the levels of consciousness, outcomes, and clinical efficacy of the treatment among patients:

#### 2.4.1 The GCS scoring scale

This is a widely used scale for measuring the level of consciousness in patients with brain injuries and comprises three components: eye‐opening response (E), verbal response (V), and motor response (M) (Teasdale & Jennett, 1974). The total score ranges from 3 (deep coma) to 15 (normal consciousness). The higher the score, the better the state of consciousness of the patient; the lower the score, the more serious the DOC (Yang et al., 2021).

#### 2.4.2 The CNPVSS scoring scale

This scale was initiated by Nanjing Zijin Hospital in 1996 to assess the state of consciousness in Chinese patients with persistent VS (PVS), which includes five items: body movement, eye movement, auditory function, eating, and emotional response (Chinese Medical Association, Brain Resuscitation Committee of Hyperbaric Medicine Branch, 2011). The total score ranges from 0 (no response) to 20 (normal response).

#### 2.4.3 GOS outcome scale

This is a widely used scale to assess the outcome of brain injury, ranging from 1 (death) to 5 (slight defect) (Jennett & Bond, 1975; Sun et al., 2021; Yuan et al., 2022). A higher score indicated a less severe craniocerebral injury. We divided the patients into poor and good prognoses groups according to the GOS scores at follow‐up, with 1–3 points reflecting a poor result and 4–5 points indicating a good outcome.

#### 2.4.4 Evaluation of clinical efficacy

We used the following criteria to evaluate the clinical efficacy of HBO treatment based on the changes in GCS and CNPVSS scores after treatment (Fan et al., 2015; Tian et al., 2012):

1. Essentially cured: The patient was sober, with a GCS score of 15 points or an overall CNPVSS score increase of more than 8 points.
2. Significant effect: Symptoms and signs were significantly improved, GCS score >12 points or CNPVSS score increased by 5–8 points.
3. Valid: Symptoms and signs were improved, GCS score >9 points, CNPVSS increased by 2–4 points.
4. Invalid: Symptoms and signs did not improve, GCS score, CNPVSS score did not increase or decrease.
5. Total effective rate = (number of basically cured cases + number of significantly effective cases + number of effective cases)/total cases × 100%.

### 2.5 Statistical methods

The experimental results were analyzed and processed by the SPSS 26.0 statistical software. In the measurement data, normally distributed data were expressed as mean ± standard deviation (x ± s), while skewed distribution data were expressed as median and quartile spacing. A paired *t*‐test or nonparametric test was used to compare the changes in GCS scores before and after treatment. A *χ* ^2^ test or rank‐sum test was used for counting data. Univariate analysis was performed on the relevant factors affecting the curative effect of HBO. Then, multivariate logistic regression analysis was performed on the significant factors; *α* = .05 was selected as the test level, and *p *< .05 was considered statistically significant.

## 3 RESULTS

### 3.1 Therapeutic effect

#### 3.1.1 GCS score

The GCS score of the patients before HBO treatment was 3–12 points, with a median and quartile of 6 (4, 8) points. After HBO treatment, the GCS score was 3–15 points, with a median and quartile of 10 (7, 13.75) points. A paired rank‐sum test was performed for GCS scores before and after HBO treatment, and the difference was statistically significant (*z* = −19.482, *p* < .01).

#### 3.1.2 CNPVSS score

The CNPVSS scores of the patients before HBO treatment were 0–8 points, and median and quartile scores were 2 (1, 2) points. After HBO treatment, the CNPVSS scores were 0–17 points, and median and quartile scores were 7 (3, 12) points. A paired rank‐sum test was performed for the CNPVSS scores before and after HBO treatment, and the difference was statistically significant (*z* = −17.651, *p* < .01).

#### 3.1.3 Evaluation of clinical efficacy

We classified the clinical efficacy into four categories based on the changes in GCS and CNPVSS scores after HBO treatment: essentially cured, significant effect, valid, and invalid. The distribution of the patients in each category was as follows: 93 of the 484 patients were essentially cured (19.2%), 83 showed significantly effective (17.1%) results, 149 reflected effective (30.8%) results, and 159 had ineffective (32.9%) results. The total effective rate was calculated as the sum of the essentially cured, significantly effective, and effective cases divided by the total cases, which was 67.1%.

### 3.2 Analysis of prognostic factors

#### 3.2.1 Single factor analysis

There were statistically significant differences between the good prognosis and the poor prognoses groups in age, HBO intervention time, HBO treatment frequency, GCS score before treatment, etiology, underlying diseases (hypertension, diabetes, coronary heart disease, and cerebrovascular disease), combined rehabilitation therapy, and HBO treatment pressure (*p* < .05) (Table 1).

**TABLE 1:** Comparison of general data of disorders of consciousness (DOC) patients with good prognosis and poor prognosis.

General information		Poor prognosis group (*n* = 139)	Good prognosis group (*n* = 298)	*χ* ^2^/*z* value	*p* value
Gender, case (%)	Male	95 (31.4%)	208 (68.6%)	0.094	.759
	Female	44 (32.8%)	90 (67.2%)		
Age, case (%)	<50 years old	63 (24.0%)	200 (76.0%)	18.782	<.001
	≥50 years old	76 (43.7%)	98 (56.3%)		
HBO intervention timing, case (%)	≤7 days	3 (7.1%)	39 (92.9%)	19.976	<.001
	8–14 days	25 (25.3%)	74 (74.7%)		
	15–30 days	63 (34.6%)	119 (65.4%)		
	>30 days	48 (42.1%)	66 (57.9%)		
HBO treatment times, case (%)	10–20 times	89 (37.1%)	151 (62.9%)	6.995	.030
	21–30 times	32 (24.4%)	99 (75.6%)		
	>30 times	18 (27.3%)	48 (72.7%)		
Pre‐treatment GCS score, *M* (P_25_, P_75_)		5 (3, 6)	7 (5, 8)	−8.542	<.001
Etiology, case (%)	Cerebral trauma	45 (20.5%)	175 (79.5%)	26.333	<.001
	Stroke	50 (43.1%)	66 (56.9%)		
	Hypoxic‐ischemic encephalopathy	44 (43.6%)	57 (56.4%)		
Hypertension, case (%)	Valid	50 (43.1%)	66 (56.9%)	9.289	.002
	N/A	89 (27.7%)	232 (72.3%)		
Diabetes mellitus, case (%)	Valid	19 (52.8%)	17 (47.2%)	7.954	.005
	N/A	120 (29.9%)	281 (70.1%)		
Coronary heart disease, case (%)	Valid	10 (71.4%)	4 (28.6%)	8.666	.003
	N/A	129 (30.5%)	294 (69.5%)		
Cerebrovascular disease, case (%)	Valid	15 (51.7%)	14 (48.3%)	5.680	.017
	N/A	124 (30.4%)	284 (69.6%)		
Combined rehabilitation therapy, case (%)	Valid	101 (37.8%)	166 (62.2%)	11.467	.001
	N/A	38 (22.4%)	132 (77.6%)		
HBO treatment of stress ①, case (%)	2.0ATA	106 (38.8%)	167 (61.2%)	16.528	<.001
	2.2ATA	33 (20.1%)	131 (79.9%)		

Abbreviations: ATA, atmospheres absolute; GCS, Glasgow Coma Scale; HBO, hyperbaric oxygen.

*Note*: ① In the HBO treatment pressure group, 2.0ATA refers to the highest pressure of 2.0ATA after the condition is stabilized, 2.2ATA refers to the highest pressure of 2.2ATA after the condition is stabilized.

#### 3.2.2 Multi‐factor analysis

Logistic multivariate regression analysis showed that late HBO intervention, fewer HBO treatments, a low GCS score before HBO treatment, and DOC caused by HIE were risk factors for a poor prognosis in patients with DOC (*p* < .05) (Table 2).

**TABLE 2:** Logistic multivariate regression analysis.

Factors	*B*	SE	Wald *χ* ^2^	*p* value	OR value	95% CI
Age	0.496	0.300	2.726	.099	1.642	0.911–2.957
HBO intervention timing						
≤7 days			26.166	<.001		
8–14 days	2.065	0.746	7.654	.006	7.886	1.826–34.062
15–30 days	2.709	0.726	13.921	<.001	15.011	3.618–62.286
>30 days	3.410	0.756	20.373	<.001	30.268	6.885‐133.069
HBO treatment times						
10–20 times			10.504	.005		
21–30 times	−0.853	0.308	7.694	.006	0.426	0.233–0.779
>30 times	−0.929	0.378	6.032	.014	0.395	0.188–0.829
Pre‐treatment GCS score	−0.477	0.068	49.718	<.001	0.621	0.544–0.709
Etiology						
Cerebral trauma			23.611	<.001		
Stroke	0.415	0.339	1.501	.220	1.515	0.780–2.943
Hypoxic‐ischemic encephalopathy	1.730	0.357	23.453	<.001	5.638	2.800–11.354
Hypertension	0.268	0.333	0.648	.421	1.307	0.681–2.511
Diabetes mellitus	0.296	0.418	0.502	.479	1.345	0.593–3.051
Coronary heart disease	−0.015	0.725	0.000	.984	0.986	0.238–4.079
Cerebrovascular disease	0.656	0.502	1.707	.191	1.928	0.720–5.161
Combined rehabilitation therapy	0.192	0.451	0.180	.671	1.211	0.500–2.934
HBO treatment stress	0.567	0.455	1.554	.213	1.762	0.723–4.297

Abbreviations: CI, confidence interval; GCS, Glasgow Coma Scale; HBO, hyperbaric oxygen; OR, odds ratio.

### 3.3 Analysis of prognostic factors of patients with DOC caused by different etiologies

#### 3.3.1 Analysis of prognostic factors of patients with DOC after traumatic brain injury

##### Univariate analysis

There were statistically significant differences in age, GCS score before HBO treatment, whether combined rehabilitation treatment was provided, and HBO treatment stress between the good prognosis and poor prognosis groups after traumatic brain injury (*p* < .05) (Table 3).

**TABLE 3:** Univariate analysis of disorders of consciousness (DOC) patients after traumatic brain injury.

General information		Poor prognosis group (*n* = 44)	Good prognosis group (*n* = 176)	*χ* ^2^ */z* value	*p* value
Causes of brain trauma, case (%)					
Car accident		42 (21.3%)	155 (78.7%)	1.626	.455
Falling		2 (9.5%)	19 (90.5%)		
Other		0 (0.0%)	2 (100.0%)		
Lesion site, case (%)					
Contusion and laceration of brain		6 (18.8%)	26 (21.3%)	6.037	.419
Subarachnoid hemorrhage		11 (21.2%)	41 (78.8%)		
Parenchymal hemorrhage		10 (32.3%)	21 (67.7%)		
Diffuse axonal injury		8 (15.7%)	43 (84.3%)		
Subdural hemorrhage		6 (22.2%)	21 (77.8%)		
Extradural hemorrhage		0 (0.0%)	9 (100.0%)		
Other		3 (16.7%)	15 (83.3%)		
Gender, case (%)	Male	29 (17.6%)	136 (82.4%)	2.424	.119
	Female	15 (27.3%)	40 (72.7%)		
Age, case (%)	<50 years old	23 (14.7%)	133 (85.3%)	9.260	.002
	≥50 years old	21 (32.8%)	43 (67.2%)		
HBO intervention timing, case (%)	≤7 days	1 (10.0%)	9 (90.0%)	2.602	.457
	8–14 days	11 (22.4%)	38 (77.6%)		
	15–30 days	17 (16.7%)	85 (83.3%)		
	>30 days	15 (25.4%)	44 (74.6%)		
HBO treatment times, case (%)	10–20 times	27 (24.1%)	85 (75.9%)	3.402	.183
	21–30 times	13 (18.6%)	57 (81.4%)		
	>30 times	4 (10.5%)	34 (89.5%)		
Pre‐treatment GCS score, *M* (P_25_, P_75_)		4.5 (3, 5.75)	7 (5, 8)	−4.913	<.001
Hypertension, case (%)	Valid	8 (30.8%)	18 (69.2%)	2.137	.144
	N/A	36 (18.6%)	158 (81.4%)		
Diabetes mellitus, case (%)	Valid	2 (40.0%)	3 (60.0%)	0.320	.572
	N/A	42 (19.5%)	173 (80.5%)		
Coronary heart disease, case (%)	Valid	0 (0.0%)	0 (0.0%)		
	N/A	44 (20.0%)	176 (80.0%)		
Cerebrovascular disease, cases (%)	Valid	2 (25.0%)	6 (75.0%)	0.000	>.999
	N/A	42 (19.8%)	170 (80.2%)		
Combined rehabilitation therapy, cases (%)	Valid	33 (27.5%)	87 (72.5%)	9.281	.002
	N/A	11 (11.0%)	89 (89.0%)		
HBO treatment stress, cases (%)	2.0ATA	33 (27.0%)	89 (73.0%)	8.506	.004
	2.2ATA	11 (11.2%)	87 (88.8%)		

Abbreviations: ATA, atmospheres absolute; GCS, Glasgow Coma Scale; HBO, hyperbaric oxygen.

##### Multiple‐*factor* analysis

Logistic regression analysis indicated that a low GCS score before HBO treatment was a risk factor for a poor prognosis among patients with DOC after a traumatic brain injury (*p* < .05) (Table 4).

**TABLE 4:** Multivariate analysis of disorders of consciousness (DOC) patients after traumatic brain injury.

Factors	*B*	SE	Wald *χ* ^2^	*p* value	OR value	95% CI
Age	0.719	0.392	3.370	.066	2.052	0.952–4.421
Pre‐treatment GCS score	−0.404	0.096	17.780	<.001	0.668	0.554–0.806
Combined rehabilitation therapy	0.570	0.638	0.797	.372	1.767	0.506–6.171
HBO treatment stress	0.447	0.635	0.495	.482	1.563	0.450–5.425

Abbreviations: CI, confidence interval; GCS, Glasgow Coma Scale; HBO, hyperbaric oxygen; OR, odds ratio.

#### 3.3.2 Prognostic factor analysis of patients with DOC after stroke

##### Univariate analysis

There were statistically significant differences in gender, HBO intervention time, HBO treatment times, and pre‐treatment GCS scores between the good and poor prognoses groups after stroke (*p *< .05) (Table 5).

**TABLE 5:** Univariate analysis of disorders of consciousness (DOC) patients after stroke.

General information		Poor prognosis group (*n* = 51)	Good prognosis group (*n* = 65)	*χ* ^2^/z value	*p* value
Causes of stroke, case (%)					
Cerebral hemorrhage		47 (43.9%)	60 (56.1%)	0.000	>.999
Cerebral infarction		4 (44.4%)	5 (55.6%)		
Lesion site, case (%)					
Basal ganglia		16 (45.7%)	19 (54.3%)	5.370	.514
Lobe		12 (40.0%)	18 (60.0%)		
Brainstem		8 (72.7%)	3 (27.3%)		
Thalamus		1 (33.3%)	2 (66.7%)		
Parencephalon		1 (33.3%)	2 (66.7%)		
Encephalocoele		1 (20.0%)	4 (80.0%)		
Other		12 (41.4%)	17 (58.6%)		
Gender, case (%)	Male	38 (51.4%)	36 (48.6%)	4.526	.033
	Female	13 (31.0%)	29 (69.0%)		
Age, case (%)	<50 years old	14 (38.9%)	22 (61.1%)	0.546	.460
	≥50 years old	37 (46.3%)	43 (53.8%)		
HBO intervention timing, case (%)	≤14 days	6 (18.8%)	26 (81.3%)	11.883	.003
	15–30 days	22 (50.0%)	22 (50.0%)		
	>30 days	23 (57.5%)	17 (42.5%)		
HBO treatment times, case (%)	10‐20 times	37 (50.0%)	37 (50.0%)	7.853	.020
	21–30 times	6 (21.4%)	22 (78.6%)		
	>30 times	8 (57.1%)	6 (42.9%)		
Pre‐treatment GCS score *M* (P_25_, P_75_)		5 (3, 6)	6 (4.5, 8)	−4.339	<.001
Hypertension, case (%)	Valid	32 (44.4%)	40 (5.6%)	0.018	.894
	N/A	19 (43.2%)	25 (56.8%)		
Diabetes mellitus, case (%)	Valid	9 (47.4%)	10 (52.6%)	0.107	.744
	N/A	42 (43.3%)	55 (56.7%)		
Coronary heart disease, case (%)	Valid	4 (57.1%)	3 (42.9%)	0.110	.740
	N/A	47 (43.1%)	62 (56.9%)		
Cerebrovascular disease, cases (%)	Valid	10 (62.5%)	6 (37.5%)	2.588	.108
	N/A	41 (41.0%)	59 (59.0%)		
Combined rehabilitation therapy, cases (%)	Valid	41 (47.7%)	45 (52.3%)	1.857	.173
	N/A	10 (33.3%)	20 (66.7%)		
HBO treatment stress, cases (%)	2.0ATA	42 (47.7%)	46 (52.3%)	2.094	.148
	2.2ATA	9 (32.1%)	19 (67.9%)		

Abbreviations: ATA, atmospheres absolute; GCS, Glasgow Coma Scale; HBO, hyperbaric oxygen.

##### Multi‐factor analysis

The logistic multivariate regression analysis demonstrated that being male, a late HBO intervention time, fewer HBO treatments, and low pre‐treatment GCS scores were risk factors for a poor prognosis among patients with DOC after stroke (*p* < .05) (Table 6).

**TABLE 6:** Multivariate analysis of disorders of consciousness (DOC) patients after stroke.

Factors	*B*	SE	Wald *χ* ^2^	*p* value	OR value	95% CI
Gender	−1.063	0.505	4.436	.035	0.345	0.128–0.929
HBO intervention timing						
≤14 days			11.910	.003		
15–30 days	1.931	0.647	8.908	.003	6.897	1.940–24.511
>30 days	2.246	0.684	10.775	.001	9.445	2.471–36.101
HBO treatments times						
10–20 times			10.044	.007		
21–30 times	−1.977	0.625	10.011	.002	0.138	0.041–0.471
>30 times	−0.433	0.711	0.371	.543	0.648	0.161–2.614
Pre‐treatment GCS score	−0.564	0.146	14.946	<.001	0.569	0.427–0.757

Abbreviations: CI, confidence interval; GCS, Glasgow Coma Scale; HBO, hyperbaric oxygen; OR, odds ratio.

#### 3.3.3 Prognostic factor analysis for patients with DOC after HIE

##### Single‐*factor* analysis

There were statistically significant differences in age, HBO intervention time, and GCS scores before HBO treatment between the good and bad prognoses groups of patients with DOC combined with HIE (*p* < .05) (Table 7).

**TABLE 7:** univariate analysis of disorders of consciousness (DOC) patients after hypoxic‐ischemic encephalopathy.

General information		Poor prognosis group (*n* = 44)	Good prognosis group (*n* = 57)	*χ* ^2^/z value	*p* value
Etiology of hypoxic ischemic encephalopathy, case (%)					
Cardiogenic cardiac arrest		21 (55.3%)	17 (44.7%)	8.860	.059
Non‐cardiac arrest		16 (48.5%)	17 (51.5%)		
Poisonous gas poisoning		3 (20.0%)	12 (80.0%)		
Drug and food poisoning		3 (42.9%)	4 (57.1%)		
Other		1 (12.5%)	7 (87.5%)		
Gender, case (%)	Male	28 (43.8%)	36 (56.3%)	0.002	.961
	Female	16 (43.2%)	21 (56.8%)		
Age, case (%)	<50 years old	26 (36.6%)	45 (63.4%)	4.689	.030
	≥50 years old	18 (60.0%)	12 (40.0%)		
HBO intervention timing, case (%)	≤7 days	2 (7.4%)	25 (92.6%)	26.150	<.001
	8–14 days	8 (34.8%)	15 (65.2%)		
	15–30 days	24 (66.7%)	12 (33.3%)		
	>30 days	10 (66.7%)	5 (33.3%)		
Number of HBO treatments, case (%)	10–20 times	25 (46.3%)	29 (53.7%)	0.400	.819
	21–30 times	13 (39.4%)	20 (60.6%)		
	>30 times	6 (42.9%)	8 (57.1%)		
Pre‐treatment GCS score, *M* (P_25_, P_75_)		4 (3, 5.75)	7 (5.5, 8)	−4.748	<.001
Hypertension, case (%)	Valid	10 (55.6%)	8 (44.4%)	1.281	.258
	N/A	34 (41.0%)	49 (59.0%)		
Diabetes mellitus, case (%)	Valid	8 (66.7%)	4 (33.3%)	2.956	.086
	N/A	36 (40.4%)	53 (59.6%)		
Coronary heart disease, case (%)	Valid	6 (85.7%)	1 (14.3%)	3.749	.053
	N/A	38 (40.4%)	56 (59.6%)		
Cerebrovascular disease, cases (%)	Valid	3 (60.0%)	2 (40.0%)	0.089	.766
	N/A	41 (42.7%)	55 (57.3%)		
Combined rehabilitation therapy, cases (%)	Valid	27 (44.3%)	34 (55.7%)	0.031	.861
	N/A	17 (42.5%)	23 (57.5%)		
HBO treatment stress, cases (%)	2.0ATA	31 (49.2%)	32 (50.8%)	2.168	.141
	2.2ATA	13 (34.2%)	25 (65.8%)		

Abbreviations: ATA, atmospheres absolute; GCS, Glasgow Coma Scale; HBO, hyperbaric oxygen.

##### Multivariate analysis

Logistic regression analysis showed that an age ≥50 years, HBO intervention timing, and low pre‐treatment GCS scores were risk factors for a poor prognosis among patients with DOC, a previous diagnosis of HIE (*p* < .05) (Table 8).

**TABLE 8:** Multivariate analysis of disorders of consciousness (DOC) patients after hypoxic ischemic encephalopathy.

Factors	*B*	SE	Wald *χ* ^2^	*p* value	OR value	95% CI
Age	1.748	0.673	6.756	.009	5.745	1.537–21.471
HBO intervention timing						
≤7 days			16.807	.001		
8–14 days	1.860	0.984	3.569	.059	6.421	0.933–44.197
15–30 days	3.533	0.974	13.166	<.001	34.229	5.077–230.793
>30 days	3.799	1.080	12.381	<.001	44.649	5.381–370.495
Pre‐treatment GCS score	−0.492	0.135	13.193	<.001	0.611	0.469–0.797

Abbreviations: CI, confidence interval; GCS, Glasgow Coma Scale; HBO, hyperbaric oxygen; OR, odds ratio.

## 4 DISCUSSION

This study analyzed the curative effect and prognostic factors of 437 patients with DOC in our department over the past 10 years. The research found that HBO therapy could improve the treatment efficacy of patients with DOC caused by different factors such as brain trauma, stroke, and HIE and increase the GCS and CNPVSS scores of patients, indicating a total effective rate of 67.1%. Through single‐factor analysis, this study found that age, the timing of HBO intervention, the frequency of HBO therapy, GCS score before HBO therapy, etiology, underlying diseases (hypertension, diabetes, coronary heart disease, and cerebrovascular disease), combined rehabilitation therapy, and the specific pressure used during HBO therapy were all influencing factors for the prognosis of patients with DOC. Further multivariate analysis revealed that a late HBO intervention time, a lower frequency of HBO therapy treatments, a low GCS score before HBO therapy, and DOC caused by HIE were independent risk factors for the poor prognosis of patients with DOC.

### 4.1 Efficacy analysis of HBO on patients with DOC

HBO therapy is used to treat diseases by inhaling pure oxygen in an environment above atmospheric pressure. In recent years, studies both at home and abroad have shown that HBO has a good therapeutic effect on DOC, which can improve the GCS, CNPVSS, and GOS scores of patients with DOC with different etiologies, improve their prognosis, and shorten coma times (Ahmadi & Khalatbary, 2021; Joshua et al., 2022; Y. S. Liu, Liu, et al., 2022; M. Liu, Li, et al., 2022; S. Y. Zhang et al., 2017). In this study, we analyzed patients with DOC who had been treated over the past 10 years. The GCS and CNPVSS scores of patients with DOC caused by brain trauma, stroke, and HIE were increased after HBO treatment, and the total effective rate was 67.1%. HBO therapy can improve brain metabolism, inhibit neuro‐inflammatory reactions, and recover nerve function, thus promoting patients to regain consciousness, shortening coma time, helping patients to recover cognitive and motor functions, and improving their daily activity abilities, thereby significantly improving the clinical efficacy and prognosis of patients (Hadanny et al., 2020; Hu et al., 2016; Li et al., 2017; X. Wang et al., 2020; W. Zhang & Zhang, 2020).

### 4.2 Analysis of prognostic factors of DOC

#### 4.2.1 Age and DOC prognosis

With increased ageing, the cerebral cortex degenerates, and the metabolic and regenerative abilities of cells in various tissue types and organs decrease to different degrees, while levels of neurotransmitters, such as acetylcholine and GABA is an important neurotransmitter in the central nervous system and has a regulatory role in a variety of body functions, also change the body (Gong et al., 2017; Guo et al., 2017). It has been noted that the total clinical response rate in patients aged <50 years is higher than in patients aged ≥50 years following HBO treatment (Pan, 2015), and that the recovery rate in patients with advanced PVS after HBO treatment is lower in patients with DOC (P. Wu et al., 2017). The univariate analysis of this study showed that the good prognosis rate of patients aged <50 years (76.0%) was higher than that of patients aged ≥50 years (56.3%), and the difference was statistically significant. However, there was no significant association between age and prognosis in patients with DOC, which is inconsistent with previous findings, possibly because the prognosis of patients is closely related to different diseases and their severity. Additionally, no direct relationship was observed between age and the severity of DOC in patients.

#### 4.2.2 Timing of HBO intervention and DOC prognosis

In the early stages of brain injury, HBO can reduce neuroinflammatory responses, improve neurological function damage, and promote the recovery of consciousness in patients (Geng et al., 2016; Y. Huang et al., 2022); in the late stages of brain injury, HBO can reduce neuroinflammatory responses by increasing arterial partial pressure of oxygen and oxygen content, improving brain tissue cell metabolism, and promoting nerve cell remodeling (Ortega et al., 2021). Studies have shown that the clinical efficacy rate of patients with DOC who started treatment 8–12 days after injury is higher than that of patients who started treatment 16–30 days after injury (Z. Huang et al., 2016). Early HBO intervention can improve the GCS and GOS scores of patients and their prognosis (Ortega et al., 2021; D. Wang et al., 2018). The multivariate analysis showed that, compared with patients whose time of HBO intervention was fewer than 7 days, the patients for whom this was 8–14 days, 15–30 days, and more than 30 days had a 6.886, 14.011, and 29.268‐fold higher rate of poor prognosis, respectively, demonstrating that an HBO intervention time ≤7 days reflected a significant improvement in patient prognosis; these results are similar to those presented in previous studies. Accordingly, when the vital signs of patients with DOC are stable, HBO therapy should be given within 7 days after the onset of the disease, as doing so can help to improve the patient's prognosis.

#### 4.2.3 Times of HBO treatment and prognosis of DOC

Multiple studies have underscored the frequency of HBO therapy as an independent factor influencing the effectiveness of treatment in patients with DOC caused by cerebral infarction (L. Liu et al., 2020), brain trauma (C. Huang & Liu, 2015; R. Wu, 2016), and HIE (Ye, 2020). Chen et al. (2021) showed that HBO therapy could improve the state of consciousness of patients with severe traumatic brain injury and suggested that patients with VS or MCS receive HBO therapy for more than 20 consecutive days. Our analysis of patients with DOC over the past decade found that the adverse prognosis rate of patients with 21–30 HBO therapy treatments and those with more than 30 treatments was 0.426 and 0.395 times higher than those with 10–20 treatments, respectively; this indicates that the prognosis of patients with 21–30 HBO treatments and those with more than 30 treatments reflected better outcomes than those with 10–20 treatments. For patients with DOC, more than 20 treatments will be helpful in terms of improving their prognosis.

#### 4.2.4 GCS score and DOC prognosis before HBO treatment

Multiple studies have shown that the lower the GCS score, the more severely damaged the brain tissue of the patient will be and, accordingly, the worse their prognosis (Zou & Luo, 2022). Additionally, a lower GCS score is a risk factor for poor prognosis in patients with DOC (Bao et al., 2021; Galea et al., 2017; Zhou, 2020). Our multivariate analysis also found that each one‐point reduction in the GCS score was associated with a 0.621‐fold increased risk of poor outcome. The lower the GCS score and the deeper the coma state of the patient at admission, the higher the risk of irreversible nerve cell damage and the worse the indicated prognosis.

#### 4.2.5 DOC etiology and prognosis

DOC can be caused by brain trauma, stroke, and HIE. The results of this study showed that, compared with HIE, patients with DOC caused by brain trauma had a better prognosis rate, and the difference was statistically significant, which is consistent with the results in other literature (Y. Liu et al., 2022). The outcomes of the present study found no statistically significant difference concerning the outcomes of patients with DOC after brain injury and stroke, however, which may be due to this having been a retrospective study; inevitably, there were confounding factors in data collection and follow‐up. The results of this study require further examination using strict and normative prospective research.

##### Analysis of prognostic factors in patients with DOC after traumatic brain injury

Through the analysis of patients with DOC arising from different causes, this study found that 80% of 220 patients with DOC caused by traumatic brain injury had a good prognosis. Multivariate analysis found that a low GCS score before HBO treatment was an independent risk factor for the poor prognosis of patients with DOC after traumatic brain injury. However, age, whether combined rehabilitation therapy was applied or not, and HBO treatment pressure settings were not identified as independent risk factors for a poor prognosis in patients with DOC after traumatic brain injury.

##### Analysis of prognostic factors in patients with DOC after stroke

In this study, 116 patients with DOC caused by stroke were analyzed. The results indicated that being male was an independent risk factor for receiving a poor prognosis among patients with DOC after stroke; the risk of a poor prognosis in men was 0.345 times higher than for women, which is inconsistent with previous research results (Xiao, 2019; Xun et al., 2021). The reason for the higher risk of an adverse prognosis in men in this study may be because the research was retrospective in nature with unbalanced gender grouping. Male participants accounted for a significant proportion of patients in this study. It is hoped that prospective studies with balanced grouping could be carried out in the future to further analyze the relationship between gender and the prognosis of patients with DOC after stroke. The multi‐factor analysis in this study also found that a late HBO intervention time, fewer HBO treatments, and low GCS scores before HBO treatment were independent risk factors for the poor prognosis of patients with DOC after stroke, which was consistent with previous studies (Alonso et al., 2015).

##### Analysis of prognostic factors in patients with DOC after HIE

In this study, 101 cases of DOC caused by HIE were analyzed; 57 cases (56.4%) had a good prognosis and 44 (43.6%) received a poor prognosis. The univariate analysis showed that age, HBO intervention timing, and GCS scores before HBO therapy were prognostic factors. Multivariate analysis showed that an age ≥50 years, delayed HBO intervention, and low GCS scores before HBO therapy were independent risk factors for poor prognosis in patients with DOC after HIE, which is consistent with previous studies (Howell et al., 2013).

## 5 CONCLUSION

This comprehensive study meticulously analyzed the influence of HBO treatment on the therapeutic outcomes of patients with DOC, employing a substantial dataset collated over a decade. The pivotal findings of this study are encapsulated below.

After HBO treatment, the GCS and CNPVSS scores of patients with DOC were significantly improved, indicating that HBO may have some beneficial effects on the treatment of DOC. However, this conclusion should be interpreted with caution, as the study design was retrospective and relied on telephone follow‐up, which may have introduced recall bias and survivorship bias. Moreover, the results did not control for potential confounding factors, such as the severity and duration of DOC, the type and extent of brain injury, and the use of other treatments. Therefore, more rigorous and prospective studies are needed to confirm the efficacy and safety of HBO for patients with DOC.

Key determinants of patient prognosis included age, the timing and frequency of HBO intervention, pre‐treatment GCS scores, and the etiology of DOC. Early HBO intervention, preferably within 7 days post‐injury, and a frequency of more than 20 treatment sessions emerged as crucial for enhancing patient prognosis. A nuanced evaluation of different etiologies revealed that for traumatic brain injury, stroke, and HIE, distinct factors, such as pre‐treatment GCS scores, gender, and age, profoundly influenced the outcomes. The insights gleaned from this study underscore the imperative for initiating HBO therapy expeditiously and tailoring the treatment frequency and duration to optimize therapeutic outcomes. The findings bolster the premise that the strategic application of HBO therapy can be pivotal in enhancing the recovery trajectory of patients with DOC. However, the study's retrospective nature necessitates further validation through prospective multi‐center studies with larger cohorts to refine and corroborate these pivotal findings and recommendations.

## AUTHOR CONTRIBUTIONS

**Sha Li**: Conceptualization; data curation; formal analysis; investigation; methodology; resources; supervision; writing—original draft. **Zhi‐Juan Di**: Conceptualization; data curation; formal analysis; methodology; project administration; resources; writing—original draft. **Zi‐Bo Liu**: Data curation; formal analysis; investigation; methodology; resources. **Long Zhao**: Data curation; formal analysis; resources; software. **Man‐Yu Li**: Data curation; formal analysis; methodology; software. **Hong‐Ling Li**: Conceptualization; data curation; formal analysis; investigation; methodology; resources; supervision; writing—review and editing.

### PEER REVIEW

The peer review history for this article is available at https://publons.com/publon/10.1002/brb3.3588

## Data Availability

All data generated or analyzed during this study are included in this published article.

## References

[brb33588-bib-0001] Chinese Medical Association, Brain Resuscitation Committee of Hyperbaric Medicine Branch . (2011). Diagnostic criteria for persistent vegetative state and clinical efficacy rating scale (China Nanjing Standard 2011 Revision). Chinese Journal of Naval Medicine and Hyperbaric Medicine, 18(5), 1.

[brb33588-bib-0002] Ahmadi, F. , & Khalatbary, A. R. (2021). A review on the neuroprotective effects of hyperbaric oxygen therapy. Medical Gas Research, 11(2), 72–82. 33818447 10.4103/2045-9912.311498 PMC8130666

[brb33588-bib-0003] Alonso, A. , Ebert, A. , Kern, R. , Rapp, S. , Hennerici, M. G. , & Fatar, M. (2015). Outcome predictors of acute stroke patients in need of intensive care treatment. Cerebrovascular Diseases, 40(1–2), 10–17. 10.1159/000430871 26022716

[brb33588-bib-0004] Bao, Y. C. , Zhang, F. , Li, Q. , Liu, M. , Cheng, X. R. , Zhang, Y. B. , & Qiu, B. (2021). [XingnaoKaiqiao acupuncture on promoting wake‐up of vegetative state after brain injury]. Zhongguo Zhen Jiu, 41(11), 1225–1228. 10.13703/j.0255-2930.20201101-k0002 34762375

[brb33588-bib-0005] Chen, W. , Jiang, Z. , Liu, S. , Huang, L. , Zhu, Q. , & Zhu, H. (2022). Effects of hyperbaric oxygen therapy on consciousness disorder and cognitive dysfunction in patients with severe traumatic brain injury. Chinese Journal of Rehabilitation Medicine, 37(10), 1326–1331+40.

[brb33588-bib-0006] Chen, Y. , Yu, H. , Ni, X. , Guo, Q. , Zheng, B. , Sun, L. , & Cao, J. (2021). Effect of hyperbaric oxygen therapy on chronic disorders of consciousness after hypoxic ischemic brain injury. Journal of Third Military Medical University, 43(15), 1449–1453.

[brb33588-bib-0007] Eapen, B. C. , Georgekutty, J. , Subbarao, B. , Bavishi, S. , & Cifu, D. X. (2017). Disorders of consciousness. Physical Medicine and Rehabilitation Clinics of North America, 28(2), 245–258. 10.1016/j.pmr.2016.12.003 28390511

[brb33588-bib-0008] Fan, F. , Meng, E. , Hu, J. , Li, N. , & Pan, Y. (2015). Hyperbaric oxygen therapy for hemorrhagic cerebral apoplexy in young adults. Journal of Translational Medicine, 4(04), 218–222.

[brb33588-bib-0009] Galea, J. P. , Dulhanty, L. , & Patel, H. C. (2017). Predictors of outcome in aneurysmal subarachnoid hemorrhage patients: Observations from a multicenter data set. Stroke, A Journal of Cerebral Circulation, 48(11), 2958–2963. 10.1161/STROKEAHA.117.017777 28974630

[brb33588-bib-0010] Geng, F. , Ma, Y. , Xing, T. , Zhuang, X. , Zhu, J. , & Yao, L. (2016). Effects of hyperbaric oxygen therapy on inflammasome signaling after traumatic brain injury. Neuroimmunomodulation, 23(2), 122–129. 27216735 10.1159/000445689

[brb33588-bib-0011] Gong, J. , Luo, C. , Feng, J. , Kong, H. , Zhanng, B. , & Liang, S. (2017). Risk factors of hip fracture in patients with acute consciousness disorder. China Medical Herald, 14(36), 80–83.

[brb33588-bib-0012] Guo, H. , Lin, H. , & Guo, J. (2017). Comparative analysis of dynamic changes of intracranial hematoma in young and old patients with acute craniocerebral injury. Chinese Journal of Clinical Neurosurgery, 22(01), 20–22.

[brb33588-bib-0013] Hadanny, A. , Rittblat, M. , Bitterman, M. , May‐Raz, I. , Suzin, G. , Boussi‐Gross, R. , Zemel, Y. , Bechor, Y. , Catalogna, M. , & Efrati, S. (2020). Hyperbaric oxygen therapy improves neurocognitive functions of post‐stroke patients—A retrospective analysis. Restorative Neurology and Neuroscience, 38(1), 93–107. 10.3233/RNN-190959 31985478 PMC7081098

[brb33588-bib-0014] Howell, K. , Grill, E. , Klein, A.‐M. , Straube, A. , & Bender, A. (2013). Rehabilitation outcome of anoxic‐ischaemic encephalopathy survivors with prolonged disorders of consciousness. Resuscitation, 84(10), 1409–1415. 23747956 10.1016/j.resuscitation.2013.05.015

[brb33588-bib-0015] Hu, S. L. , Feng, H. , & Xi, G. H. (2016). Hyperbaric oxygen therapy and preconditioning for ischemic and hemorrhagic stroke. Medical Gas Research, 6(4), 232–236. 10.4103/2045-9912.196907 28217297 PMC5223316

[brb33588-bib-0016] Huang, Z. , Fu, Y. , Li, Z. , & Wang, C. (2015). Long‐term efficacy and related factors of hyperbaric oxygen therapy for severe craniocerebral trauma. Guide of China Medicine, 13(26), 145–146.

[brb33588-bib-0017] Huang, Y. , Xiao, F.‐M. , Tang, W.‐J. , Qiao, J. , Wei, H.‐F. , Xie, Y.‐Y. , & Wei, Y. (2022). Hydrogen inhalation promotes recovery of a patient in persistent vegetative state from intracerebral hemorrhage: A case report and literature review. World Journal of Clinical Cases, 10(4), 1311–1319. 10.12998/wjcc.v10.i4.1311 35211564 PMC8855194

[brb33588-bib-0018] Huang, Z. , Fu, Y. , & Li, Z. (2016). Comparison of timing and efficacy of hyperbaric oxygen intervention in patients with craniocerebral trauma. Hainan Medical Journal, 27(19), 3161–3163.

[brb33588-bib-0019] Hyldegaard, O. , & Hedetoft, M. (2020). [Hyperbaric oxygen therapy]. Ugeskrift for Laeger, 182(44), V06200463 (in Danish). 33118490

[brb33588-bib-0020] Jennett, B. , & Bond, M. (1975). Assessment of outcome after severe brain damage. Lancet, 1(7905), 480–484. 46957 10.1016/s0140-6736(75)92830-5

[brb33588-bib-0021] Joshua, T. G. , Ayub, A. , Wijesinghe, P. , & Nunez, D. A. (2022). Hyperbaric oxygen therapy for patients with sudden sensorineural hearing loss: A systematic review and meta‐analysis. JAMA Otolaryngology—Head & Neck Surgery, 148(1), 5–11. 10.1001/jamaoto.2021.2685 34709348 PMC8554691

[brb33588-bib-0022] Kondziella, D. , Bender, A. , Diserens, K. , Van Erp, W. , Estraneo, A. , Formisano, R. , Laureys, S. , Naccache, L. , Ozturk, S. , Rohaut, B. , & Sitt, J. D. (2020). European Academy of Neurology guideline on the diagnosis of coma and other disorders of consciousness. European Journal of Neurology, 27(5), 741–756. 32090418 10.1111/ene.14151

[brb33588-bib-0023] Li, X. , Li, J. , Yang, X. , Sun, Z. , Zhang, J. , Zhao, W. , Dong, S. , Li, C. , Ye, Y. , Chen, J. , Li, Y. , Xiang, Y. , Mao, J. , Li, G. , Guo, H. , Zhang, W. , Guo, H. , Zhang, Y. , Zhang, M. , … Wang, Y. (2017). Hyperbaric‐oxygen therapy improves survival and functional outcome of acute severe intracerebral hemorrhage. Archives of Medical Research, 48(7), 638–652. 10.1016/j.arcmed.2018.03.001 29548729

[brb33588-bib-0024] Liu, L. , Yu, H. , Wang, C. , & Xue, B. (2020). Hyperbaric oxygen therapy for cerebral infarction after clipping unruptured intracranial aneurysm and its influencing factors analysis. Chinese Journal of Stroke, 15(08), 842–847.

[brb33588-bib-0025] Liu, M. , Li, Q. , Bao, Y. , Ma, Y. , Niu, Y. , & Zhang, F. (2022). Effect of low frequency repetitive transcranial magnetic stimulation (rTMS) combined with hyperbaric oxygen (HBO) on awakening of coma patients with traumatic brain injury. Journal of Healthcare Engineering, 2022, 6133626. 10.1155/2022/6133626 35449850 PMC9018176

[brb33588-bib-0026] Liu, Y. , Kang, G. , Liu, Y. , Song, G. , & Jiang, W. (2022). Establishment of long‐term prognosis model in patients with chronic consciousness disorder. Journal of Air Force Medical University, 43(09), 967–972.

[brb33588-bib-0027] Liu, Y. S. , Liu, Z. B. , Yang, Z. , Zhao, L. , & Li, H. L. (2022). Clinical efficacy of hyperbaric oxygen combined with different timings of right median‐nerve electrical stimulation in patients with brain injury‐induced disorders of consciousness. Brain and Behavior, 12(9), e2716. 10.1002/brb3.2716 35920129 PMC9480931

[brb33588-bib-0028] Marcinkowska, A. , Mankowska, N. D. , Kot, J. , & Winklewski, P. J (2022). Impact of hyperbaric oxygen therapy on cognitive functions: A systematic review. Neuropsychology Review, 32(1), 99–126. 33847854 10.1007/s11065-021-09500-9 PMC8888529

[brb33588-bib-0029] Ortega, M. A. , Fraile‐Martínez, O. , García‐Montero, C. , Callejón‐Peláez, E. , Saez, M. A. , Alvarez‐Mon, M. A. , García‐Honduvilla, N. , Monserrat, J. , Alvarez‐Mon, M. , & Buján, J. (2021). A general overview on the hyperbaric oxygen therapy: Applications, mechanisms and translational opportunities. Medicina, 57(9), 864. 10.3390/medicina57090864 34577787 PMC8465921

[brb33588-bib-0030] Pan, W. (2015). Clinical effect evaluation of hyperbaric oxygen for cerebral resuscitation after cardiopulmonary resuscitation. China Health Standard Management, 6(28), 53–54.

[brb33588-bib-0031] Song, M. , Yang, Y. , Yang, Z. , Cui, Y. , Yu, S. , He, J. , & Jiang, T. (2020). Prognostic models for prolonged disorders of consciousness: An integrative review. Cellular and Molecular Life Sciences, 77(20), 3945–3961. 32306061 10.1007/s00018-020-03512-z PMC11104990

[brb33588-bib-0032] Sun, L. , Liu, C. , Wang, Q. , Yang, H. , Di, B. , & Steven, L. (2021). Frontier progress: Exploring the path of consciousness disorder research and transformation in China from an international perspective. Health Research, 41(05), 485–489.

[brb33588-bib-0033] Teasdale, G. , & Jennett, B. (1974). Assessment of coma and impaired consciousness. A practical scale. Lancet, 2(7872), 81–84. 4136544 10.1016/s0140-6736(74)91639-0

[brb33588-bib-0034] Tian, W. , Wang, M. , & Sun, L. (2012). Clinical observation of continuous vegetative state induced by comprehensive rehabilitation. Chinese Journal of Rehabilitation Medicine, 27(04), 283–285.

[brb33588-bib-0035] Wang, D. , Liu, G. , & Yang, S. (2018). Effect of different timing of hyperbaric oxygen therapy on the curative effect of patients with moderate and severe craniocerebral trauma. Practical Journal of Cardiac Cerebral Pneumal and Vascular Diseases, 26(01), 138–140.

[brb33588-bib-0036] Wang, X. , Chen, Y. , Wang, Z. , & Qian, M. (2020). Clinical research of early hyperbaric oxygen therapy on patients with hypertensive cerebral hemorrhage after craniotomy. Turkish Neurosurgery, 30(3), 361–365. 10.5137/1019-5149.JTN.25044-18.3 30984995

[brb33588-bib-0037] Wang, Q. , Xia, T. , Yang, Q. , Yu, S. , & Cheng, C. (2015). Effect of stroke unit combined with hyperbaric oxygen on consciousness disorder of acute cerebral hemorrhage. Chinese Journal of Nautical Medicine and Hyperbaric Medicine, 22(06), 459–461+66.

[brb33588-bib-0038] Wu, R. (2016). Long‐term efficacy and influencing factors of hyperbaric oxygen therapy for severe craniocerebral trauma. World Latest Medicine Information, 16(A0), 114–116.

[brb33588-bib-0039] Wu, P. , Chen, Y. , Yan, H. , Huang, J. , Gui, G. , Wu, M. , Zhang, F. , & Xiao, H. (2017). Survival status and influencing factors of patients with persistent vegetative state. Chinese General Practice, 20(05), 558–562.

[brb33588-bib-0040] Xiao, S. (2019). Study on the risk factors and prognosis of ischemic stroke . Peking Union Medical College.

[brb33588-bib-0041] Xun, K. , Mo, J. , Ruan, S. , Dai, J. , Zhang, W. , Lv, Y. , Du, N. , Chen, S. , Shen, Z. , & Wu, Y. (2021). A meta‐analysis of prognostic factors in patients with posterior circulation stroke after mechanical thrombectomy. Cerebrovascular Diseases, 50(2), 185–199. 10.1159/000512609 33378751

[brb33588-bib-0042] Yang, Y. , Dong, J. , Hu, J. , & Yu, W. (2021). Analysis of PICC catheter‐associated infection in patients with long‐term consciousness disturbance and intervention measures. Chinese Journal of Nautical Medicine and Hyperbaric Medicine, 28(1), 47–50.

[brb33588-bib-0043] Ye, J. (2020). Application of hyperbaric oxygen in the treatment of cerebral resuscitation. Jilin University.

[brb33588-bib-0044] Yuan, C. , Dong, W. , & Pan, T. F (2022). Relationship between the level of serum D‐dimer and the severity and prognosis of hypertensive cerebral hemorrhage patients. Medical Information, 35(13), 165–167.

[brb33588-bib-0045] Zhang, S. Y. , Liu, S. B. , Xie, H. W. , Chen, Y. M. , Liao, K. L. , Xiang, Y. , & Pan, D. (2017). [“Xingnao Kaiqiao” acupuncture(acupuncture technique for restoring consciousness) combined with hyperbaric oxygen in patients with hypoxic ischemic encephalopathy: Clinical observation and its influence on blood rheology]. Zhen Ci Yan Jiu, 42(6), 518–521. 10.13702/j.1000-0607.2017.06.010 29318859

[brb33588-bib-0046] Zhang, W. , & Zhang, B. (2020). Recent advances in the protective mechanism of hyperbaric oxygen therapy for ischemic stroke. Journal of Cardio‐Cerebrovascular Diseases of Traditional Chinese and Western Medicine, 18(15), 2451–2453.

[brb33588-bib-0047] Zhang, Y. , Zhou, Y. , Jia, Y. , Wang, T. , & Meng, D. (2023). Adverse effects of hyperbaric oxygen therapy: A systematic review and meta‐analysis. Frontiers in Medicine, 10, 1160774. 37275378 10.3389/fmed.2023.1160774 PMC10232961

[brb33588-bib-0048] Zhao, J. Z. (2020). Advancement and current status of clinical diagnosis and treatment of consciousness disorders. Journal of Clinical Neurosurgery, 17(01), 1–3+7.

[brb33588-bib-0049] Zhou, Y. (2020). Effect of hyperbaric oxygen combined with functional rehabilitation training on long‐term prognosis and influencing factors of patients with severe craniocerebral trauma. Heilongjiang Medical Journal, 44(07), 916–918.

[brb33588-bib-0050] Zou, T. , & Luo, J. (2022). Effect of hyperbaric oxygen therapy on patients with persistent vegetative state after craniocerebral injury. China Medical Herald, 19(05), 89–92.

